# Unraveling the significance of AGPAT4 for the pathogenesis of endometriosis via a multi-omics approach

**DOI:** 10.1007/s00439-024-02681-2

**Published:** 2024-06-08

**Authors:** Jun Chen, Licong Shen, Tingting Wu, Yongwen Yang

**Affiliations:** 1 Department of Infectious Diseases, Xiangya Hospital, Central South University, Changsha, 410008 China; 2 Department of Gynecology, Xiangya Hospital, Central South University, Changsha, 410008 China; 3 Department of Cardiovasology, Xiangya Hospital, Central South University, Changsha, 410008 China; 4 Department of Clinical Laboratory, Xiangya Hospital, Central South University, No. 87 Xiangya Road, Changsha, 410008 China; 5 National Clinical Research Center of Geriatric Disorders, Xiangya Hospital, Central South University, Changsha, 410008 China

## Abstract

Endometriosis is characterized by the ectopic proliferation of endometrial cells, posing considerable diagnostic and therapeutic challenges. Our study investigates AGPAT4’s involvement in endometriosis pathogenesis, aiming to unveil new therapeutic targets. Our investigation by analyzing eQTL data from GWAS for preliminary screening. Subsequently, within the GEO dataset, we utilized four machine learning algorithms to precisely identify risk-associated genes. Gene validity was confirmed through five Mendelian Randomization methods. AGPAT4 expression was measured by Single-Cell Analysis, ELISA and immunohistochemistry. We investigated AGPAT4’s effect on endometrial stromal cells using RNA interference, assessing cell proliferation, invasion, and migration with CCK8, wound-healing, and transwell assays. Protein expression was analyzed by western blot, and AGPAT4 interactions were explored using AutoDock. Our investigation identified 11 genes associated with endometriosis risk, with AGPAT4 and COMT emerging as pivotal biomarkers through machine learning analysis. AGPAT4 exhibited significant upregulation in both ectopic tissues and serum samples from patients with endometriosis. Reduced expression of AGPAT4 was observed to detrimentally impact the proliferation, invasion, and migration capabilities of endometrial stromal cells, concomitant with diminished expression of key signaling molecules such as Wnt3a, β-Catenin, MMP-9, and SNAI2. Molecular docking analyses further underscored a substantive interaction between AGPAT4 and Wnt3a.Our study highlights AGPAT4’s key role in endometriosis, influencing endometrial stromal cell behavior, and identifies AGPAT4 pathways as promising therapeutic targets for this condition.

## Introduction

Endometriosis, a prevalent benign gynecological condition, affects approximately 10% of women in their reproductive years (Szukiewicz 2023; Zieliński et al. 2023). There are three forms of endometriosis: superficial, ovarian, and deep infiltrating (Rzewuska et al. 2023). This condition leads to a range of debilitating symptoms including chronic pelvic pain, painful menstruation, deep-seated discomfort, challenges with urination, extreme fatigue, and infertility. It significantly impacts patients’ physical health, mental and emotional well-being, sexual health, social interactions, and overall productivity (Nassiri Kigloo et al. 2023; Song et al. 2023). Despite its benign nature, endometriotic cells share several characteristics with malignant cancer cells, notably their migratory and invasive capabilities (Yan et al. 2020). Various hypotheses have been proposed to explain the development of endometriosis, yet its exact etiology and pathogenesis remain elusive. The predominant theory suggests that endometriotic tissue originates from retrograde menstruation (Lamceva et al. 2023; Muraoka et al. 2023). Although menstrual retrograde is a common physiological phenomenon, and endometriosis occurs only in a minority of women, other factors must also contribute. Consequently, endometriosis may develop due to these factors adhering to ovaries, ligaments, and peritoneal surfaces (Shi et al. 2023). Recent research indicates that ectopic endometrium cells, which diverge from normal endometrial cells, could play a beneficial role in promoting the growth, attachment, and longevity of endometrial tissue within the peritoneal cavity of individuals suffering from endometriosis (Izumi et al. 2023). Furthermore, recent findings highlight the significant role of the Wnt signaling pathway in endometrial stromal cells (ESCs) from women with endometriosis (Zhang et al. 2023). Specifically, the Wnt/β-catenin pathway may be instrumental in promoting the regeneration and mesenchymal transition of the endometrium.

Recent studies reveal a significant link between endometriosis and lipid metabolism, suggesting that disruptions in lipid metabolic pathways could be integral to understanding and potentially treating the disease (Yang et al. 2022; Dai et al. 2023). AGPAT4, integral to the AGPAT family, orchestrates lipid metabolism by facilitating the conversion of lysophosphatidic acid to phosphatidic acid, essential in triglyceride and phospholipid synthesis (Du et al. 2022; Pan et al. 2024). This enzyme’s influence transcends its metabolic role, as it is entwined with diverse biological processes and diseases, particularly cancer, where its dysregulation correlates with tumor growth and metastasis (Zhukovsky et al. 2019). The connection of AGPAT4 with significant oncological metrics such as histological grading, lymphatic dissemination, and prognosis highlights its potential as both a biomarker and a therapeutic target (Basili et al. 2020).The role of AGPAT4 in lipid regulation also ties it to metabolic anomalies, pointing to a broader impact on cellular functions and disease etiology (Du et al. 2022). In the context of endometriosis, the function of AGPAT4 is yet to be fully unraveled.

This study leverages an integrative approach, employing expression quantitative trait loci (eQTL) data from genome-wide association studies (GWAS) and the Gene Expression Omnibus (GEO) database analyzed through advanced R software and machine learning algorithms, to dissect the role of AGPAT4 in endometriosis. By elucidating the mechanistic pathways and biological impacts of AGPAT4, this research aims to underscore its therapeutic promise in mitigating endometriosis, potentially revolutionizing treatment paradigms.

## Materials and methods

### Participant recruitment and sample gathering

We integrated eQTL data encompassing 19,942 genes from the GWAS catalog as the exposure factor, alongside endometriosis datasets ebi-a-GCST90018839 and ukb-d-IBD_ENDOMETRIOSIS, boasting extensive sample populations of 231,771 and 361,194, respectively. To fortify our analysis, we included transcriptomic data from GSE7305, GSE11691, GSE23339, and GSE25268, forming a composite validation cohort of 79 subjects, delineated into 57 endometriosis cases and 22 controls. Further depth was added through GSE214411, which provided single-cell profiles from 128,243 endometrial cells across ten subjects, including six with minimal/mild endometriosis and four controls.

Ectopic and corresponding eutopic endometrial specimens were meticulously collected from patients diagnosed with ovarian endometriotic cysts during laparoscopic surgeries conducted at the Gynecological Department of Xiangya Hospital, within the timeframe of January 2022 to October 2023. Histological examinations post-surgery confirmed the diagnosis of endometriosis. Control specimens were similarly sourced from individuals presenting with benign ovarian cysts unrelated to endometriosis. The study encompassed 38 endometriosis patients with an average age of 31.0 ± 4.4 years, and a control group of 43 individuals with an average age of 29.0 ± 3.2 years. All participants were characterized by regular menstrual cycles and had refrained from hormonal treatments in the three months preceding their surgeries. The timing of sample collection was strategically aligned with the proliferative phase of the menstrual cycle, as corroborated by preoperative assessments and histopathological evaluations. Informed consent was diligently obtained from all participants, and the study protocol received ethical clearance from the Medical Ethics Committee of Xiangya Hospital, Central South University, under approval number 202,109,936.

### Elucidating endometriosis risk genes via integrative mendelian randomization, machine learning, and single-cell transcriptomics

We utilized a quintet of methodologies—Inverse Variance Weighted, Weighted Median, MR Egger, Weighted Mode, and Simple Mode—leveraging the TwoSampleMR package to discern endometriosis-associated risk genes (Bowden et al. 2015). We focused on genes from the ebi-a-GCST90018839 and ukb-d-IBD_ENDOMETRIOSIS datasets exhibiting odds ratios (OR) greater than 1, and identified common risk genes through their intersection. Subsequent to batch correction and normalization, transcriptome datasets from GSE7305, GSE11691, GSE23339, and GSE25268 were amalgamated using the sva package to forge a composite validation cohort. Within this cohort, the 11 identified risk genes underwent further scrutiny through machine learning algorithms—Random Forest (RF), Support Vector Machine (SVM), Extreme Gradient Boosting (XGB), and Generalized Linear Model (GLM)—with their efficacy evaluated via Receiver Operating Characteristic (ROC) curves.

Following machine learning validation, the risk genes’ cellular localization was elucidated using single-cell data from GSE214411, adhering to established protocols primarily involving the Seurat and SingleR packages (Fonseca et al. 2023). The integrity of the Mendelian Randomization (MR) outcomes was rigorously assessed through leave-one-out sensitivity analysis and the construction of funnel plots, ensuring the robustness and reliability of our findings in elucidating the genetic underpinnings of endometriosis.

### Immunohistochemistry

For the immunohistochemical quantification of AGPAT4, we prepared 4 μm thick paraffin-embedded tissue sections, which were subjected to standard deparaffinization and rehydration protocols. Antigen retrieval was facilitated through microwave heating. To quench endogenous peroxidase activity, sections were immersed in 3% hydrogen peroxide for 10 min. Subsequently, the sections were incubated with a polyclonal rabbit anti-AGPAT4 antibody (Abmart, TD3640, 1:200) at 4 °C overnight, followed by a 30-minute incubation at room temperature with a horseradish peroxidase-conjugated secondary antibody targeting rabbit immunoglobulins. Visualization was achieved using diaminobenzidine (DAB) staining, counterstained with hematoxylin, and the sections were then dehydrated and mounted under coverslips. Imaging was performed with a Leica Upright Metallurgical Microscope (Wetzlar, Germany).

The evaluation of AGPAT4 expression involved assessing both the staining intensity and the proportion of positive cells. Staining intensity was categorized as 0 (no staining), 1 (weak), 2 (moderate), or 3 (strong), and the percentage of AGPAT4-positive cells was scored as 0 (none), 1 (≤ 25%), 2 (> 25% to < 50%), or 3 (≥ 50%) (Akbar et al. 2015). The immunoreactive score was determined by multiplying the intensity of staining by the percentage of positively stained cells.

### Enzyme-linked immunosorbent assay

In the ELISA validation cohort, we included 38 endometriosis patients and 43 control subjects. Serum levels of AGPAT4 were quantitatively determined employing an ELISA kit (Abmart, TD3640, China) with a dilution of 1:2000, adhering strictly to the provided manufacturer’s protocol. The optical density at 450 nm (OD_450_), indicative of AGPAT4 concentration, was measured utilizing a microplate reader (Infinite M200 PRO, TECAN) subsequent to the application of the colorimetric substrate.

### Cell isolation and culture

ESCs were cultured from eutopic endometrial samples of women with endometriosis. Samples were collected under aseptic conditions, washed, and transported on ice. ESCs were isolated, passaged using standard trypsinization, and cultivated in phenol red-free DMEM supplemented with 10% FBS at 37 °C with 5% CO_2_. ESC purity was validated through vimentin immunostaining (Abcam), and only cultures with a purity exceeding 95% were considered for inclusion in the study (Canosa et al. 2017).

### Transfection experiments

In this investigation, RNA interference was executed via small interfering RNA (siRNA) transfection technique. Targeting the AGPAT4 gene, three distinct siRNAs were synthesized by Ribo Bio, China, supplemented with a control siRNA for comparative purposes. A cohort of 10^4 ESCs were seeded in six-well plate for 24 h prior to the transfection procedure. The transfection process involved both the AGPAT4-specific and control siRNAs using the riboFECT mRNA Transfection Reagent, procured from Ribo Bio, in strict adherence to the manufacturer’s guidelines. Subsequent to a 72-hour incubation post-transfection, cellular samples were subjected to Western blot analysis to ascertain the efficacy of gene suppression. Additional assays were conducted at 48 h following the harvesting of the cells.

### Western blotting

Western blotting was performed in accordance with standard protocols. Briefly, proteins were extracted from lysed cells using a radioimmunoprecipitation assay buffer and then clarified by centrifugation at 12,000×g for 15 min at 4 °C. Protein levels in the supernatant were quantified using the bicinchoninic acid method (Themofisher). The proteins were then resolved by electrophoresis on 10% SDS-polyacrylamide gels and transferred to polyvinylidene difluoride membranes (Millipore Billerica). Membranes were blocked with 5% non-fat milk before being incubated with primary antibodies targeting GAPDH (Proteintech, 80570-1-RR,1:10,000), β-Catenin (Cell Signaling Technology, D10A8,1:2,000), MMP-9 (Proteintech, 10375-2-AP, 1:1,000), Wnt3a (Sangon Biotech, D122111,1:2,000), SNAI2 (Sangon Biotech, D221235,1:5,000), and AGPAT4 (Abmart, TD3640, 1:2,000). Following incubation with horseradish peroxidase-conjugated secondary antibodies (goat anti-rabbit) at room temperature for one hour, the blots were washed and developed. Protein bands were quantified using Quantity One software, with GAPDH serving as the normalization control.

### Cell proliferation assay

The proliferative capacity of ESCs was evaluated using a Cell Adhesion assay, with a focus on their adhesion characteristics. This assessment was conducted via a CCK-8-based assay, executed in a 96-well plate format. To facilitate the assay, each well was treated with 50 µL of Matrigel, diluted in a serum-free medium at a 1:8 ratio. Following a 48-hour transfection period, a density of 4 × 10^3 cells per 200 µL was cultured in each well, which then underwent an incubation phase for 30 min. Subsequent to this incubation, non-adherent cells were gently washed away. Thereafter, each well received 20 µL of CCK-8 reagent (Biosharp, BS350A, China), followed by an additional incubation period of four hours. The adhesion efficiency of the ESCs was quantitatively analyzed by measuring the optical density (OD) at 450 nm using a spectrophotometric plate reader, with the OD values serving as an indicator of the number of adherent cells.

### Wound-healing assay

To assess cellular migratory capabilities, a wound-healing assay was conducted on cells transfected with AGPAT4-specific siRNA. Cells were first cultured in six-well plates to achieve 90% confluency. Subsequently, a standardized wound was introduced into the cellular monolayer using a 200 µL plastic pipette tip. Post-wounding, the cells were rinsed with phosphate-buffered saline (PBS) to remove any detached cellular debris. Digital images of each well were captured at two time points: immediately after wounding (0 h) and 48 h post-wounding. The width of the wound was quantified utilizing Image-Pro Plus software. The migration rate was calculated using the formula: [Cell-free area at 0 h - Cell-free area at 48 h] / Cell-free area at 0 h, effectively measuring the reduction in wound width over the 48-hour period.

### Transwell invasion assay

To determine the invasive potential of ESCs, a transwell invasion assay was meticulously conducted. The preparatory phase involved coating the upper chamber of the transwell setup with 60 µL of Matrigel, prepared at a 1:2 ratio with DMEM lacking phenol red, followed by an incubation period of one hour at 37 °C. Subsequently, ESCs were seeded into these upper chambers at a density of 10^3 cells per well. The lower chambers were supplemented with DMEM devoid of phenol red, enriched with 10% fetal bovine serum (FBS). Post a 72-hour incubation interval, cells residing in the upper chamber were carefully removed. The transwell filters underwent fixation using 4% paraformaldehyde for 30 min, followed by a double washing in phosphate-buffered saline (PBS). The staining process involved 0.5% hematoxylin, applied for a duration of 5 min. The invasive cells were then enumerated in three distinct fields, using a Leica Upright Metallurgical Microscope (Wetzlar, Germany). To quantify the invasion, the absorbance at 550 nm was measured utilizing a spectrophotometric plate reader. To bolster the experimental validity, this entire procedure was replicated thrice.

### Molecular docking of AGPAT4 and Wnt3a

To investigate the interaction and structural relationship between AGPAT4 and Wnt3a, this study employed a high-precision molecular docking approach. Initially, the protein structures of AGPAT4 and Wnt3a were obtained from the RCSB PDB database (https://www.rcsb.org/). Subsequently, using the Auto-dock software, we conducted ten independent molecular docking simulations to ensure the reliability and accuracy of our results. This method allowed us to analyze the potential interactions between AGPAT4 and Wnt3a at a molecular level in detail.

### Statistical analysis

The GEO data was subjected to rigorous analysis using R software (v4.2.1), adhering to the standards of robust data processing. Statistical computations and inferential analyses were conducted using SPSS software, version 22.0 (SPSS, Inc., Chicago, USA), a staple in quantitative research. Descriptive statistics are presented as mean ± standard deviation, providing a clear understanding of data variability and central tendency. The one-way Analysis of Variance (ANOVA) was the chosen statistical method to discern the differences among multiple experimental groups. A threshold of *P* < 0.05 was set for statistical significance, ensuring that the results were statistically robust and reliable. This level of significance was meticulously maintained throughout the analysis to uphold the integrity of the statistical findings. All experiments were conducted in duplicate.

## Results

### Potential risk genes for endometriosis

**Fig. 1:**
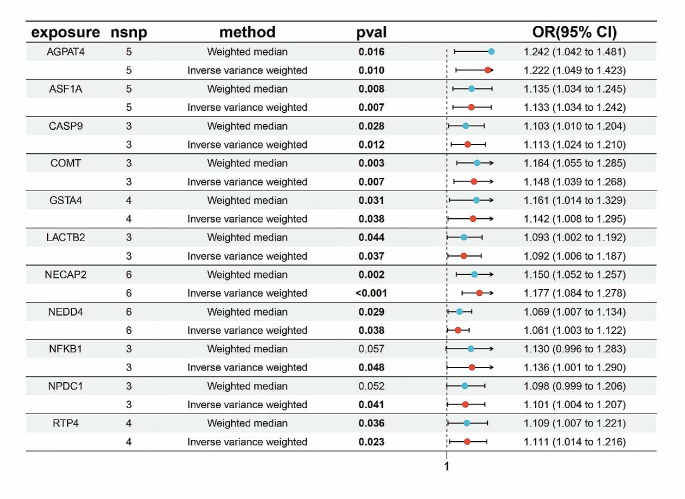
Mendelian Randomization eQTL Risk Gene Forest Plot for Endometriosis. nsnp: the number of single nucleotide polymorphisms, pval: *p* value, OR: odds ratio, 95%CI: 95% confidence interval

**Fig. 2:**
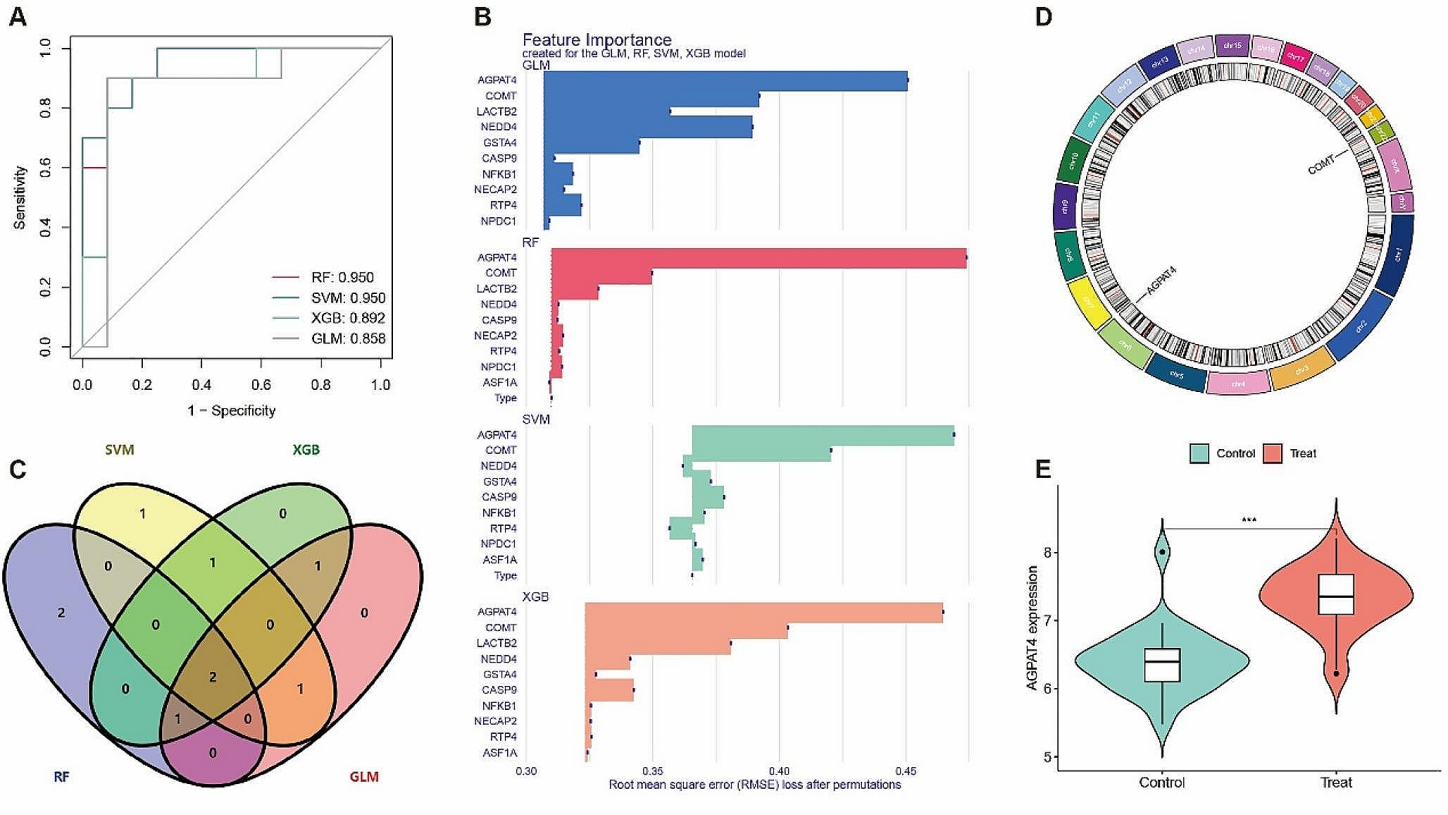
Machine learning to screen for risk genes. (**A**) Diagnostic efficacy of four machine learning models. (**B**) Key genes identified by the four machine learning models. (**C**) Venn diagram of risk genes validated by the four machine learning models. (**D**) Chromosomal localization of AGPAT4 and COMT. (**E**) Expression levels of the AGPAT4 gene in the validation dataset. Control: Normal endometrial tissue, Treat: Endometriosis lesion tissue. *p < 0.05, **p < 0.01, ***p < 0.001

**Fig. 3:**
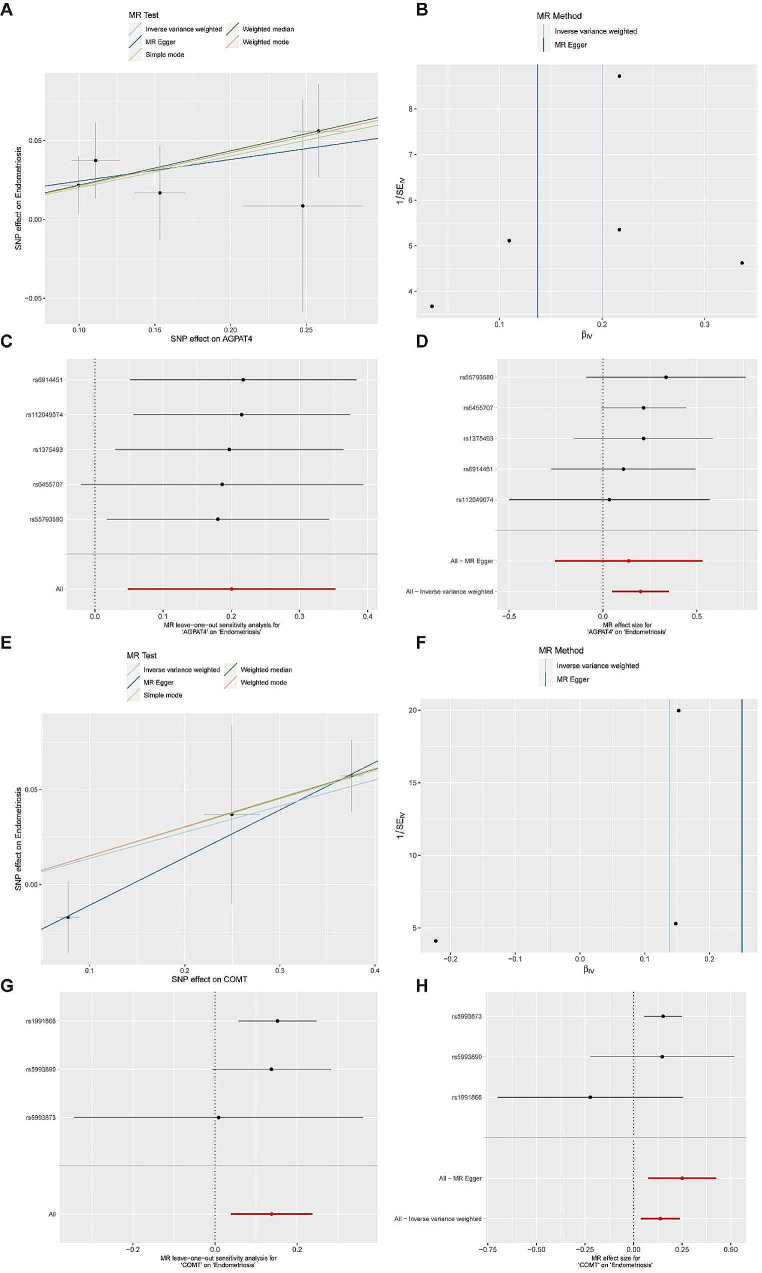
MR Results and sensitivity analysis of AGPAT4 and COMT. (**A**) Scatter Plot of Endometriosis and AGPAT4 Association via Five Mendelian Randomization Analysis Methods. (**B**) MR Funnel Plot for AGPAT4 and Endometriosis Association. (**C**) leave one out sensitivity analysis for AGPAT4 on Endometriosis. (**D**) Forest Plot of MR Effect Size for AGPAT4 on Endometriosis. (**E**) Scatter Plot of Endometriosis and COMT Association via Five Mendelian Randomization Analysis Methods. (**F**) MR Funnel Plot for COMT and Endometriosis Association. (**G**) leave one out sensitivity analysis for COMT on Endometriosis. (**H**) Forest Plot of MR Effect Size for COMT on Endometriosis

Through Mendelian randomization, we identified 11 risk genes for endometriosis, visualized using forest plots (Fig. 1). Subsequently, four machine learning algorithms were employed for validation (Fig. 2-A), with the GLM algorithm showing the lowest AUC of 0.858. The top ten genes from each machine learning model were visualized (Fig. 2-B). Intersection analysis of the top five ranked genes based on important scores across all four machine learning models (Fig. 2-C) revealed the presence of AGPAT4 and COMT in all models. Gene localization identified their positions on human chromosomes 6 and 22, respectively (Fig. 2-D). AGPAT4 was significantly upregulated in the validation dataset (Fig. 2-E). Five Mendelian randomization methods indicated that both AGPAT4 and COMT could serve as risk genes for endometriosis. The reliability of MR results was assessed using leave-one-out sensitivity analysis and funnel plots (Fig. 3), enhancing the credibility of our findings.

### Elevated AGPAT4 expression in endometriosis

**Fig. 4:**
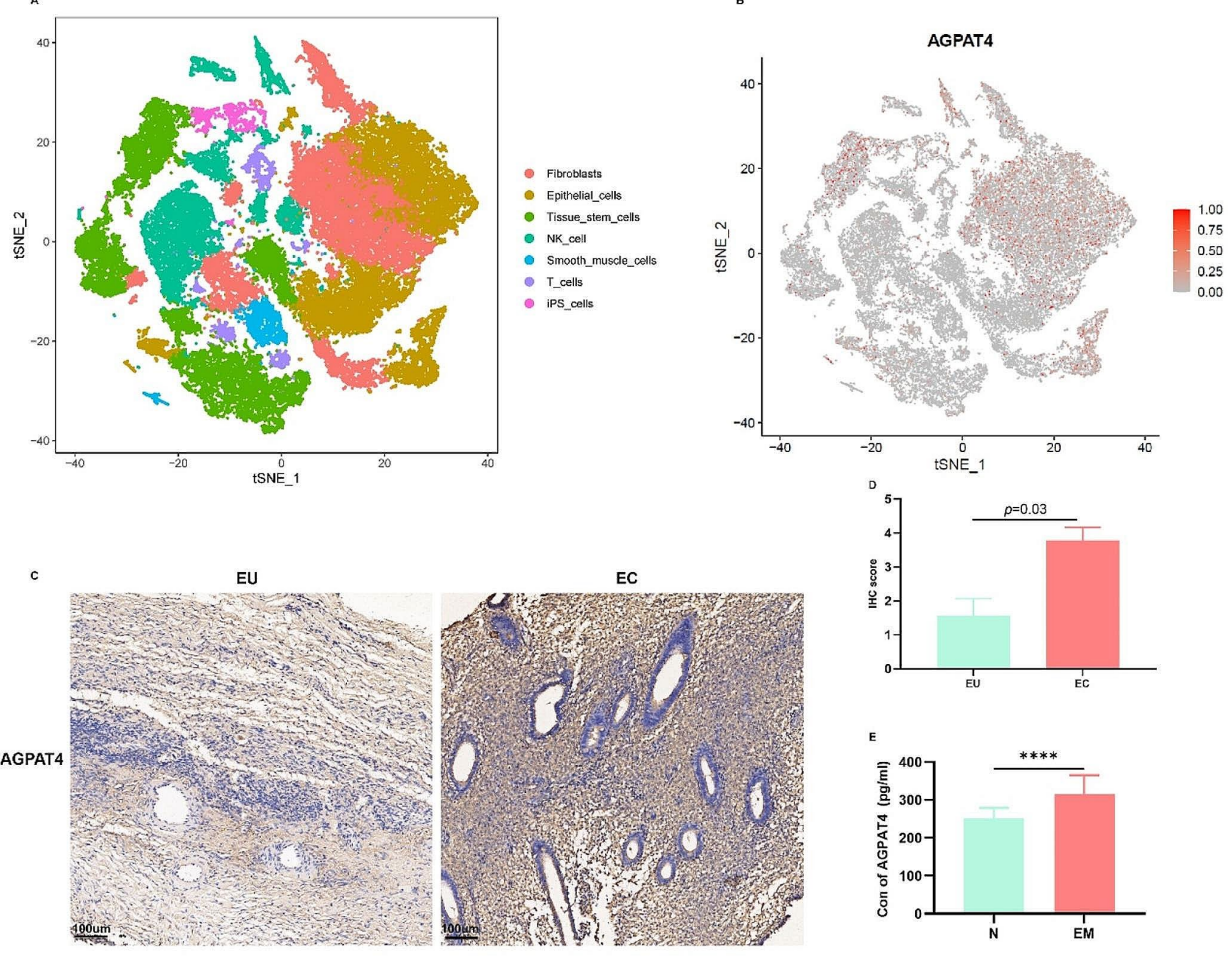
AGPAT4 Expression Levels in GSE214411, Peripheral Blood Plasma, and Tissues. (**A**) Major cellular composition of the endometrium in GSE214411. (**B**) Distribution of AGPAT4 expression across various cell types in GSE214411. (**C**) AGPAT4 expression in ectopic versus eutopic endometrial tissues. AGPAT4 expression in brown and nucleus in blue. (**D**) Comparative scores of AGPAT4 expression in ectopic and eutopic endometrial tissues. (**E**) AGPAT4 expression levels in peripheral blood of controls versus endometriosis patients. N: normal people, EM: patients with endometriosis, EU: eutopic endometrial tissues, EC: ectopic endometrial tissues. *p < 0.05, **p < 0.01, ***p < 0.001

Single-cell data analysis revealed that AGPAT4 is primarily expressed in the epithelial cells and tissue stem cells of the endometrium (Fig. 4A-B). Immunohistochemical analysis of tissues confirmed AGPAT4’s presence in both epithelial and stromal cells, with a predominant cytoplasmic localization (Fig. 4C). Notably, AGPAT4 protein expression in the ectopic endometrium of ovarian endometriosis was markedly elevated compared to the eutopic tissue group (*P* < 0.05, Fig. 4D). Plasma levels of the AGPAT4 protein in endometriosis patients were significantly higher compared to the control group (*P* < 0.01) (Fig. 4E). Unfortunately, the results for COMT were not significant in our clinical samples.

### AGPAT4 knockdown suppressed the proliferation of ESCs

**Fig. 5:**
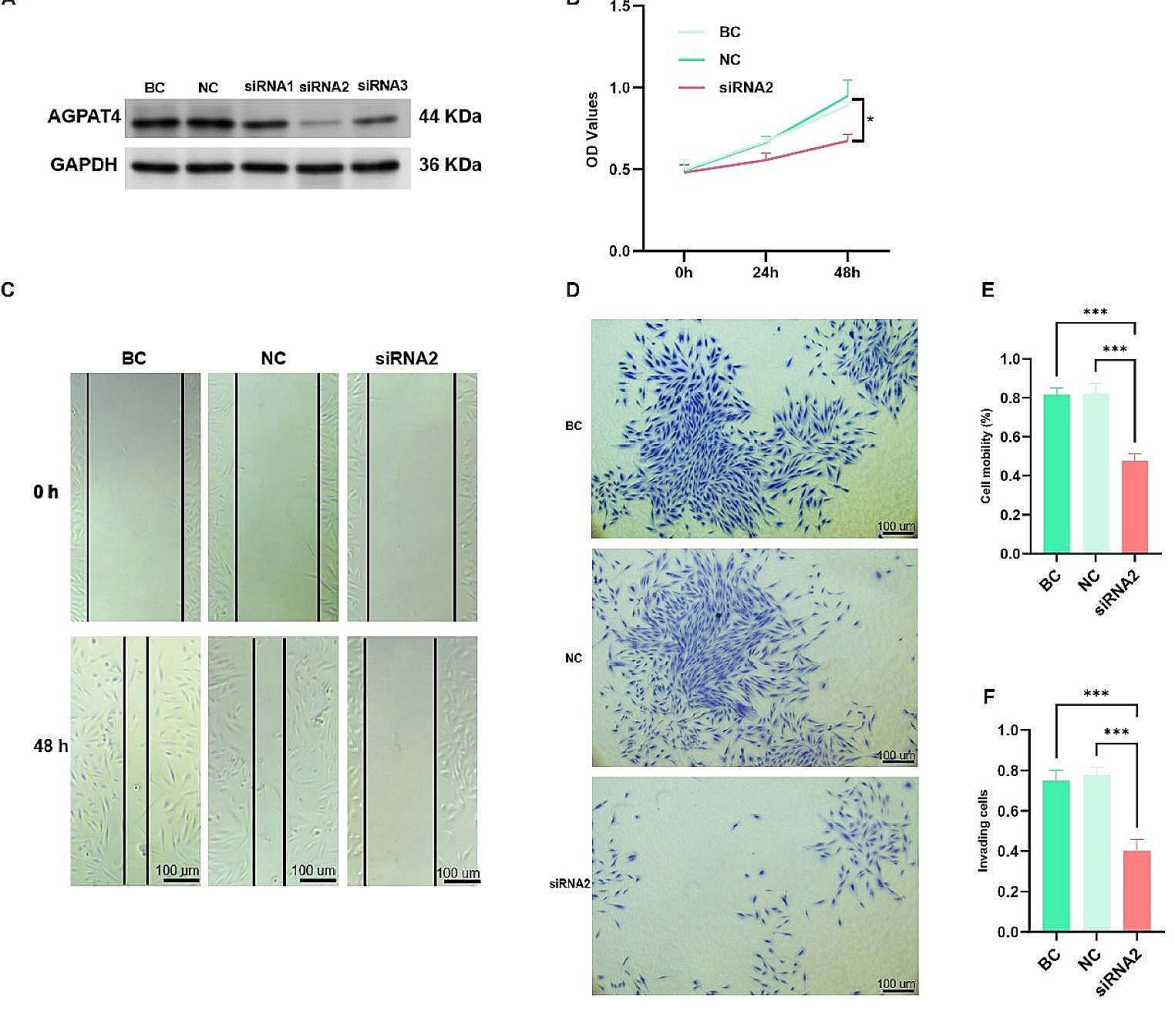
The effect of AGPAT4 on the proliferation, migratory and invasive capabilities of ESCs. (**A**) Down-regulation of AGPAT4 assessed by western blotting after transfection with three short interfering RNA (siRNA) or the negative control (NC). The second siRNA (siRNA2) was selected for further investigations. BC, blank control without siRNA. (**B**) Knockdown of AGPAT4 had negative effect on the proliferation of ESCs. (**C**-**D**) Knockdown of AGPAT4 had positive effect on the migratory of ESCs. (**E**-**F**) Down-regulation of AGPAT4 inhibited the invasive capability of ESCs. ESCs: endometrial stromal cells. *p < 0.05, **p < 0.01, ***p < 0.001

ESCs were subjected to transfection using three distinct siRNA sequences aimed at targeting AGPAT4. Subsequent Western blot analysis revealed a notable downregulation of AGPAT4 protein levels in cells transfected with the si-AGPAT4-2 sequence, as compared to those in the negative (NC) and blank (BC) control groups (Fig. 5A). Given the efficacy observed, siRNA2 was selected for subsequent experiments.

To elucidate the role of AGPAT4 in the proliferation dynamics of ESCs, cells were transfected with either siRNA2 or siNC for a duration of 48 h. Post-transfection, a Cell Counting Kit-8 (CCK-8) assay was employed to assess cellular proliferation. The assay outcomes demonstrated that silencing AGPAT4 via siRNA2 transfection significantly impeded the proliferation of ESCs, as evidenced by the comparative analysis with the NC and BC groups (*p* < 0.05, Fig. 5B).

### Suppression of AGPAT4 attenuates migration and invasion capabilities in ESCs

To elucidate the influence of diminished AGPAT4 expression on the migratory and invasive behaviors of ESCs, comprehensive analyses were conducted utilizing both wound-healing and transwell assays. The results, illustrated in Fig. 5C and F, indicated a pronounced decline in the migration and invasion capacities of ESCs following AGPAT4 knockdown. This decrease was statistically significant when juxtaposed against the outcomes observed in the NC and BC groups (*p* < 0.01), underscoring the pivotal role of AGPAT4 in modulating these critical cellular functions.

### Impact of AGPAT4 on key molecule expression in ESCs

**Fig. 6:**
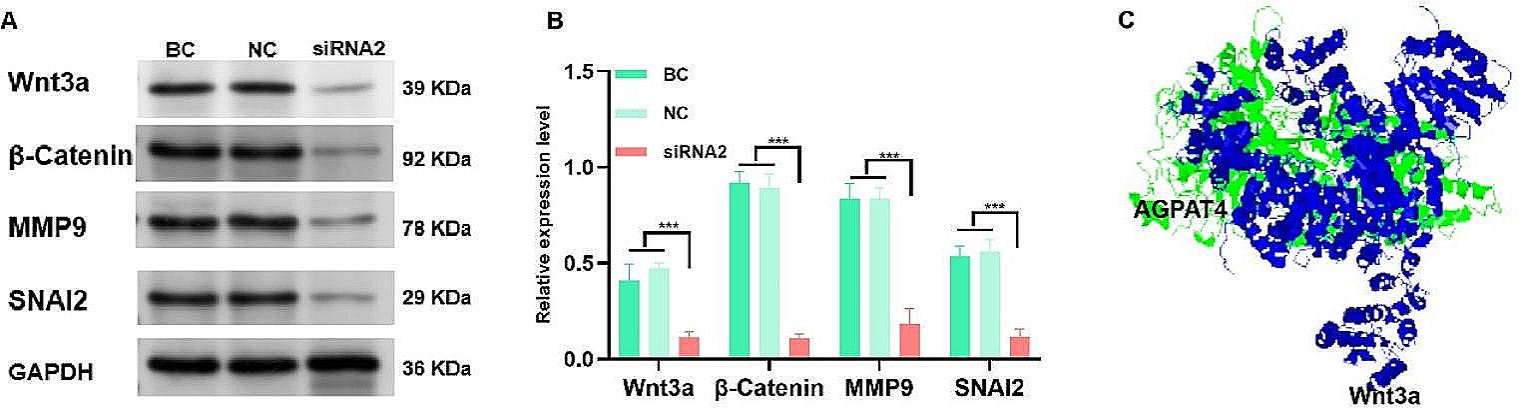
Effects of AGPAT4 down-regulation on related molecule expression. (**A**) Representative western blots analysis, with values normalized to GAPDH. (**B**) Quantification results of AGPAT4 down-regulation on related molecule expression (Wnt3a,β-Catenin, MMP9,SNAI2). (**C**) Molecular docking of AGPAT4 and Wnt3a. BC: Blank control, NC: Negative control. *p < 0.05, **p < 0.01, ***p < 0.001

To determine the influence of AGPAT4 on key molecules associated with cell proliferation and invasion, specifically the Wnt3a/β-Catenin pathway and migration and invasion-related molecules MMP-9 and SNAI2, ESCs were analyzed post-transfection with siRNA2 for 72 h. Western blot analysis was employed for this assessment. The findings revealed that the downregulation of AGPAT4 notably reduced the expression levels of Wnt3a, β-Catenin, MMP-9, and SNAI2 in ESCs, when compared to both BC and NC groups. This decrease was statistically significant, as evidenced by the data presented in Fig. 6A and B (*p* < 0.01), highlighting the regulatory role of AGPAT4 in these critical molecular pathways in endometrial stromal cells. After conducting ten simulation molecular docking analyses using Auto-Dock, it was discovered that the interaction between AGPAT4 and Wnt3a, as illustrated in Fig. 6C. The interface of AGPAT4 and Wnt3a was characterized by several hydrogen bonds and hydrophobic interactions. Specifically, key residues in AGPAT4, such as Arginine 100 and Lysine 150, form hydrogen bonds with Aspartate 45 and Threonine 50 of Wnt3a, respectively. Additionally, the hydrophobic patch around Leucine 115 of AGPAT4 engages with the hydrophobic core of Wnt3a, enhancing the stability of the complex.

## Discussion

Endometriosis emerges as a multifaceted clinical syndrome, the genesis of which is intertwined with an array of genetic and environmental determinants. In the realm of contemporary research, the integration of GWAS and eQTL data through Mendelian Randomization has been pivotal in demystifying the genetic underpinnings of a spectrum of complex diseases and clinical phenomena (Porcu et al. 2019; Gleason et al. 2021). The advent of machine learning methodologies has further revolutionized the analysis of extensive genetic and transcriptomic datasets, facilitating the discernment of intricate patterns and correlations within the vast expanse of bioinformatics data (Li et al. 2023; Ren et al. 2024). This confluence of Mendelian Randomization, eQTL analytics, and advanced computational algorithms has significantly augmented our capacity to decode the genetic and molecular frameworks of endometriosis, thereby propelling our understanding of its pathophysiology to new horizons (de Leeuw et al. 2022; Li et al. 2023).

Our Mendelian randomization analysis, leveraging eQTL data, has significantly advanced our understanding of the genetic landscape of endometriosis by identifying 11 risk genes. The gene’s significance was corroborated using advanced machine learning techniques, notably SVM, RF, XGB, and GLM (Greener et al. 2022). These models, each with distinctive attributes and broad applicability within machine learning disciplines (Sherkatghanad et al. 2023), were selectively employed based on the dataset’s unique characteristics, ensuring rigorous validation against GEO dataset benchmarks. The discovery that AGPAT4 is predominantly expressed in epithelial and tissue stem cells, as revealed by single-cell transcriptomics (Fonseca et al. 2023), coupled with its elevated expression in the peripheral blood plasma of individuals with endometriosis and its pronounced presence in ectopic versus eutopic tissues. Silencing AGPAT4 in cancer cell lines led to inhibited tumor growth in various models, suggesting that AGPAT4 might influence cancer progression through tumor microenvironment modulation ​ (Zhang et al. 2020). However, the mechanism of action of AGPAT4 in endometriosis is unknown.

The siRNA-mediated silencing of AGPAT4 resulted in a marked inhibition of interstitial cell proliferation, migration, and invasion, accompanied by the downregulation of key signaling molecules such as Wnt3a, MMP9, SNAIL, and β-Catenin. Wnt3a stands as a central figure in this interplay, acting as the principal activator in the Wnt signaling pathway (Peña-Oyarzún et al. 2023). Its activation is crucial for kickstarting a cascade of events essential for cellular proliferation and differentiation (Sukarawan et al. 2023). This process leads to the stabilization and accumulation of β-Catenin, a protein that plays dual roles in gene transcription regulation and cell-cell adhesion (Wang et al. 2023).MMP-9, a key member of the matrix metalloproteinases family, plays an indispensable role in the degradation of extracellular matrix components (Liu et al. 2023). This function is crucial not only for normal tissue remodeling and cellular migration but also in pathological states such as cancer (Coates-Park et al. 2023). Our findings also highlight its importance in endometriosis. Adding to this complex interplay is SNAI2, a transcription factor that plays a crucial role in orchestrating cell differentiation and migration (Zhang et al. 2023). The expression of SNAI2, regulated by the Wnt/β-Catenin signaling pathway (Duan et al. 2022), forms a direct link between the intracellular signaling mechanisms and the transcriptional control of genes essential for cell motility and invasion (Ottone et al. 2023).These findings not only highlight the critical role of AGPAT4 in the pathophysiology of endometriosis but also suggest its potential as a therapeutic target, particularly in light of molecular docking studies indicating a possible interaction between AGPAT4 and Wnt3a​​. Research indicates that the Wnt/β-Catenin signaling pathway plays a crucial role in regulating lipid metabolism and is implicated in various disease states including obesity, non-alcoholic fatty liver disease, and cancer progression (Bagchi et al. 2020; Liu et al. 2022; Zheng et al. 2022). This potential interaction may represent a direct molecular link, influencing the Wnt signaling pathway and thereby affecting critical downstream cellular processes, including proliferation and differentiation. Interestingly, our study did not reveal significant expression of COMT, another gene previously implicated in endometriosis, suggesting a more prominent role for AGPAT4 in the disease’s pathogenesis (Zhang et al. 2020). This highlights the complexity of endometriosis and underscores the need for a multifaceted approach to unravel its molecular basis.

## Conclusion

Our research not only elucidates the genetic underpinnings of endometriosis but also positions AGPAT4 as a central figure in potential therapeutic strategies. Future research should focus on further elucidating the molecular interactions and functional roles of AGPAT4, paving the way for innovative treatments that could offer relief to millions affected by this debilitating condition.

## Data Availability

No datasets were generated or analysed during the current study.
